# Management of a Pediatric Oral Hemangioma Using Intralesional Sodium Tetradecyl Sulfate: A Case Report

**DOI:** 10.1002/ccr3.70905

**Published:** 2025-09-22

**Authors:** Bibek Kattel, Niroj Khanal, Rojeena Adhikari, Sushma Jaishi, Pramodman Singh Yadav

**Affiliations:** ^1^ College of Dental Surgery BP Koirala Institute of Health Sciences Dharan Nepal; ^2^ Department of Oral and Maxillofacial Surgery Rapti Academy of Health Sciences Dang Nepal; ^3^ Department of Orthodontics and Dentofacial Orthopedics College of Medical Sciences Bharatpur Nepal; ^4^ BP Koirala Institute of Health Sciences Dharan Nepal

**Keywords:** benign vascular lesion, oral hemangioma, pediatric hemangioma, sclerotherapy, sodium tetradecyl sulfate

## Abstract

Benign vascular lesions called oral hemangiomas are frequently seen in young children. In this case study, intralesional sodium STS was used successfully to treat a 10‐year‐old girl who had an oral hemangioma. The lesion was noted a few weeks after birth and was located on maxillary labial and vestibular mucosa from teeth 12 to 16, which has effaced the right nasolabial fold. Diagnosis was confirmed through ultrasonography and contrast‐enhanced computer tomography (CECT) imaging. The patient had intralesional STS injections several times, which significantly decreased the size of the lesion and eliminated the related symptoms. The effectiveness of STS as a non‐surgical therapy option for children's oral hemangiomas is demonstrated by this report.

**Summary**

- Intralesional sodium tetradecyl sulfate (STS) can be an effective non‐surgical treatment for oral hemangiomas in children. This case of a 10‐year‐old girl demonstrated significant lesion reduction and symptom relief with repeated STS injections, highlighting its value as a minimally invasive therapeutic option.

## 1 Introduction

In children, especially in newborns, oral hemangiomas are frequent vascular lesions. Usually appearing in the first few weeks of infancy, these lesions sometimes don't cause any symptoms until later in life. Depending on where they occur in the oral cavity, hemangiomas can appear superficial or deep. They are often made up of groups of blood vessels. Despite their benign nature, they may result in bleeding difficulties, functional disruptions, or aesthetic issues [1, 2].

Some hemangiomas may remain or even expand, requiring intervention, even though many of them undergo natural involution. There are various therapeutic options available, like corticosteroid injection, sclerotherapy, laser therapy, and surgical excision [3, 4, 5].

Among these, intralesional injection of STS has become a commonly performed non‐surgical treatment for oral hemangiomas. STS causes localized thrombosis and inflammation in the blood vessels, which results in fibrosis and reduces the size of the lesion [3, 4].

This case shows significant efficacy of intralesional STS in shrinking oral hemangiomas, which benefits the pediatric patient population in functional and aesthetic aspects. It provides valuable insights into the technical approach, number of sessions required, and the excellent long‐term functional and aesthetic outcomes achieved, offering a strong non‐surgical alternative for similar cases.

## 2 Case Presentation

A 10‐year‐old female presented to the Department of Dentistry at Rapti Academy of Health Science (RAHS) with her parents with the chief complaint of bluish swelling on the right side of her upper jaw that had been there since she was a baby. A few months after the baby was born, the mother first noticed the lesion, which was very small and asymptomatic. But with time, the lesion increased progressively. The child had little masticatory difficulties because of the size and position of the swelling.

**FIGURE 1:**
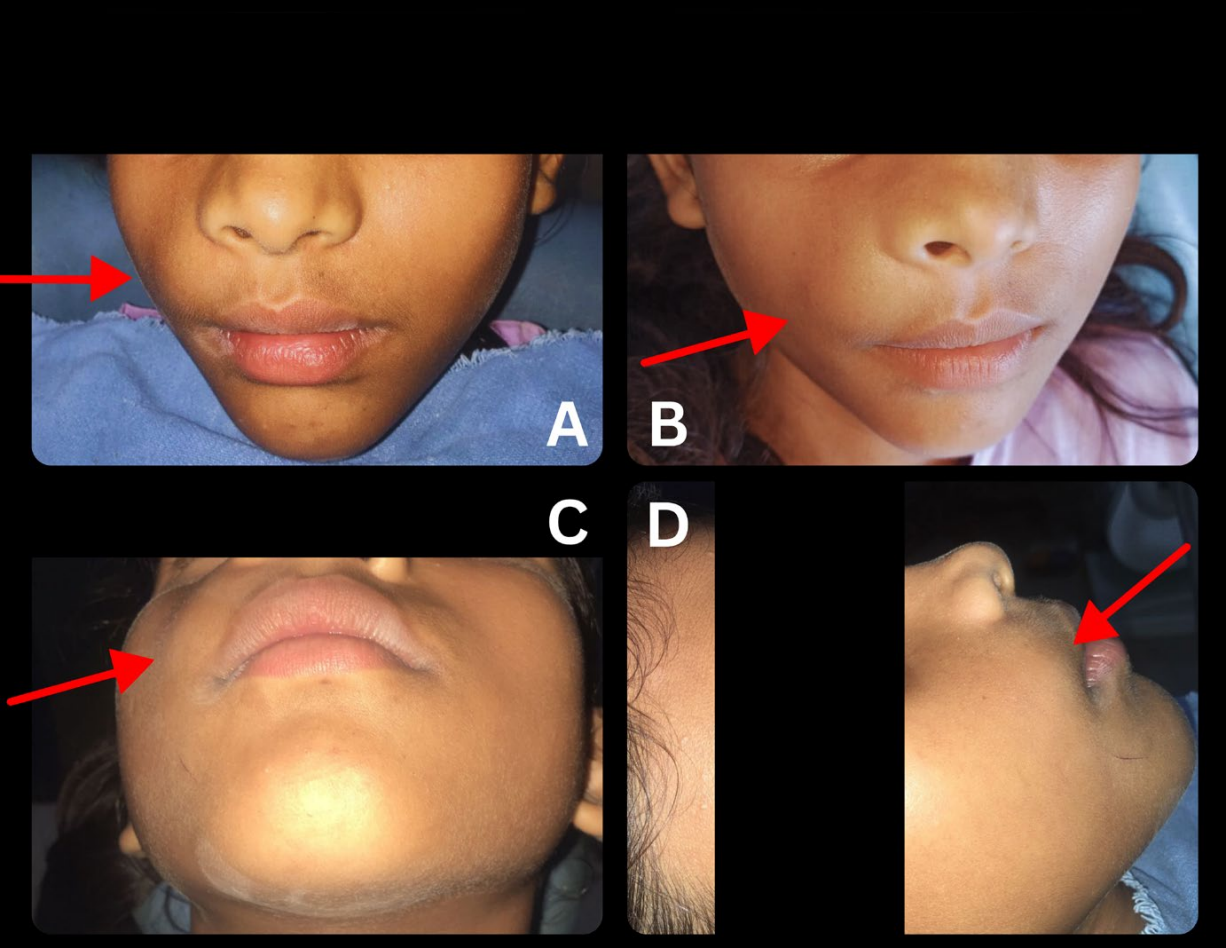
Pre‐treatment extraoral photographs showing facial swelling in the maxillary right labial mucosa and vestibular region with slight elevated alae of nose: (A and B) Frontal view, (C) Worm's eye view/submento‐vertical view (D) Right lateral profile view. The area of interest is indicated by a red arrowhead.

**FIGURE 2:**
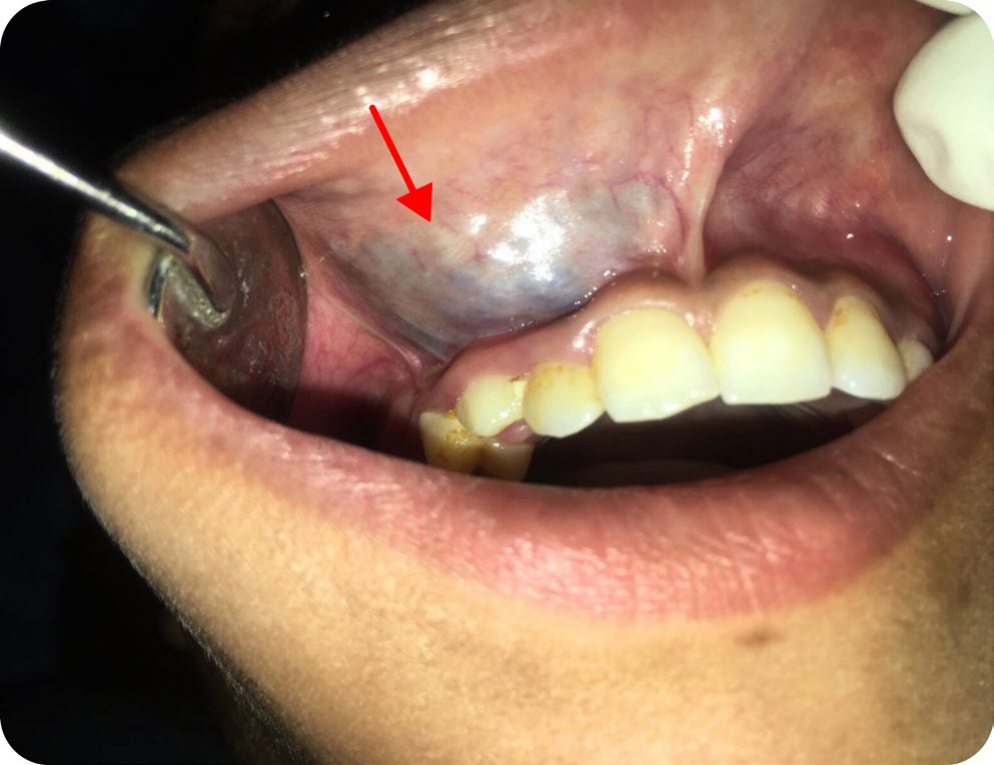
Pre‐treatment Intraoral photograph showing the extension of the lesion with bluish hue extending from lateral incisor to first molar of first quadrant. The area of interest is indicated by a red arrowhead.

Clinical examination revealed a distinct, non‐tender, compressible enlargement in the maxillary right labial mucosa and vestibule with slight elevation of the alae of the nose (Figure 1). Intraorally, the lesion extends from the lateral incisor to the first molar of the first quadrant (Figure 2). Auscultation revealed no bruits, thrills, or pulsation, ruling out the likelihood of an arteriovenous malformation with no sign of ulceration.

**FIGURE 3:**
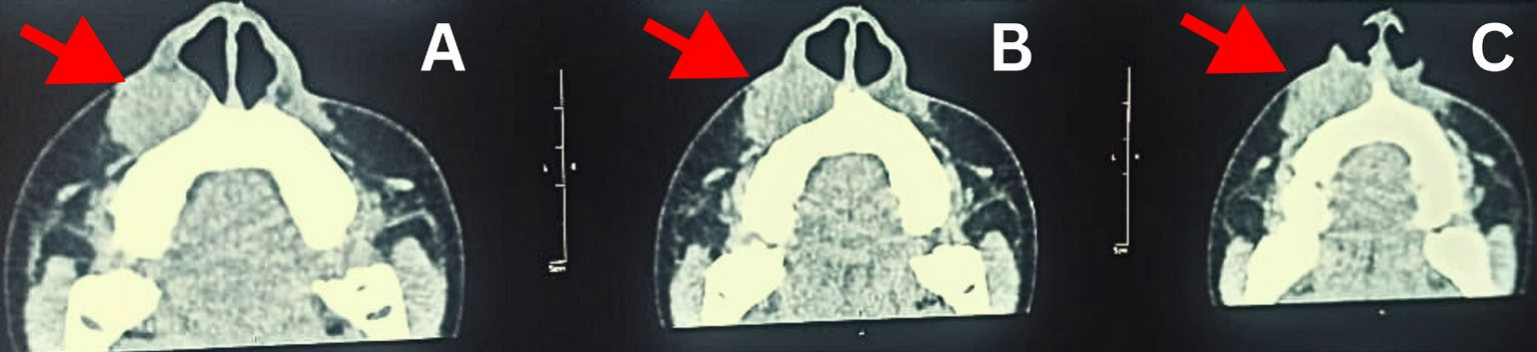
CT images showing well defined soft tissue lesion in right ala‐facial region (A), (B), (C) showing extension of the lesion. The area of interest is indicated by a red arrowhead.

Color Flow Doppler ultrasonography showed clear vascular flow and characteristics similar to a hemangioma. The diagnosis was verified by contrast‐enhanced computed tomography of the maxilla, which revealed a distinct vascular abnormality affecting the mucosa and vestibular mucosa (Figure 3). No significant systemic illnesses were elicited. Laboratory tests such as hemogram, liver function tests, and renal function tests, Prothrombin time/International normalized ratio (PT/INR), bleeding time (BT), clotting time (CT), and serology were all within normal ranges.

## 3 Treatment Plan and Procedure

Since the lesion was benign, a non‐surgical method was used. Intralesional STS, a sclerosing drug well known for its efficacy in treating vascular abnormalities, was administered to the patient [3, 5].

### 3.1 First Visit

**FIGURE 4:**
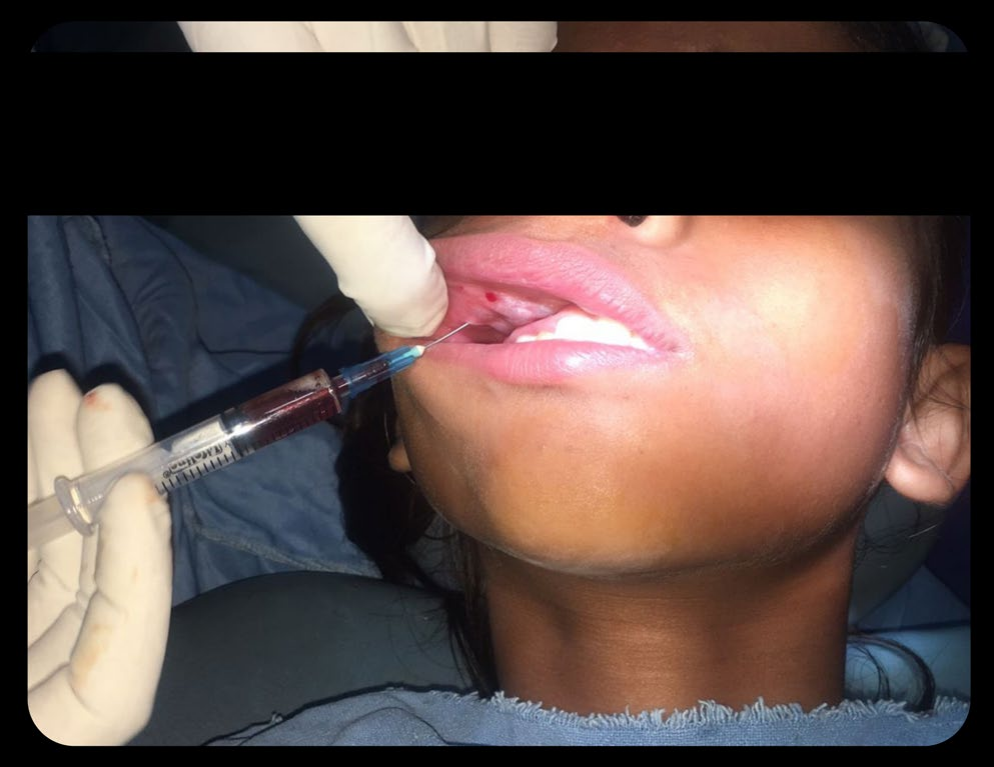
Administration of Intralesional Sclerosing agent (STS). The area of interest is indicated by a red arrowhead.

Under sterile conditions, the infraorbital nerve block was given through the maxillary incisal approach using 2% lignocaine with 1:200,000 adrenaline. Two milliliters of 60 mg/2 ml STS were injected around the lesion using a 27‐gauge needle. Four injection sites were selected to guarantee even distribution of the sclerosing agent. The needle was inserted to the lesion from healthy mucosa (approximately 5 mm). Positive aspiration ensured that we were in the lesion. 0.5 ml of solution was deposited in each site (Figure 4). To facilitate the sclerosing agent's absorption and action, the lesion was physically squeezed after injection. For the first 24 h following the procedure, the patient was provided 250 mg of paracetamol three times a day.

### 3.2 Second Visit (Two‐Week Post Treatment)

**FIGURE 5:**
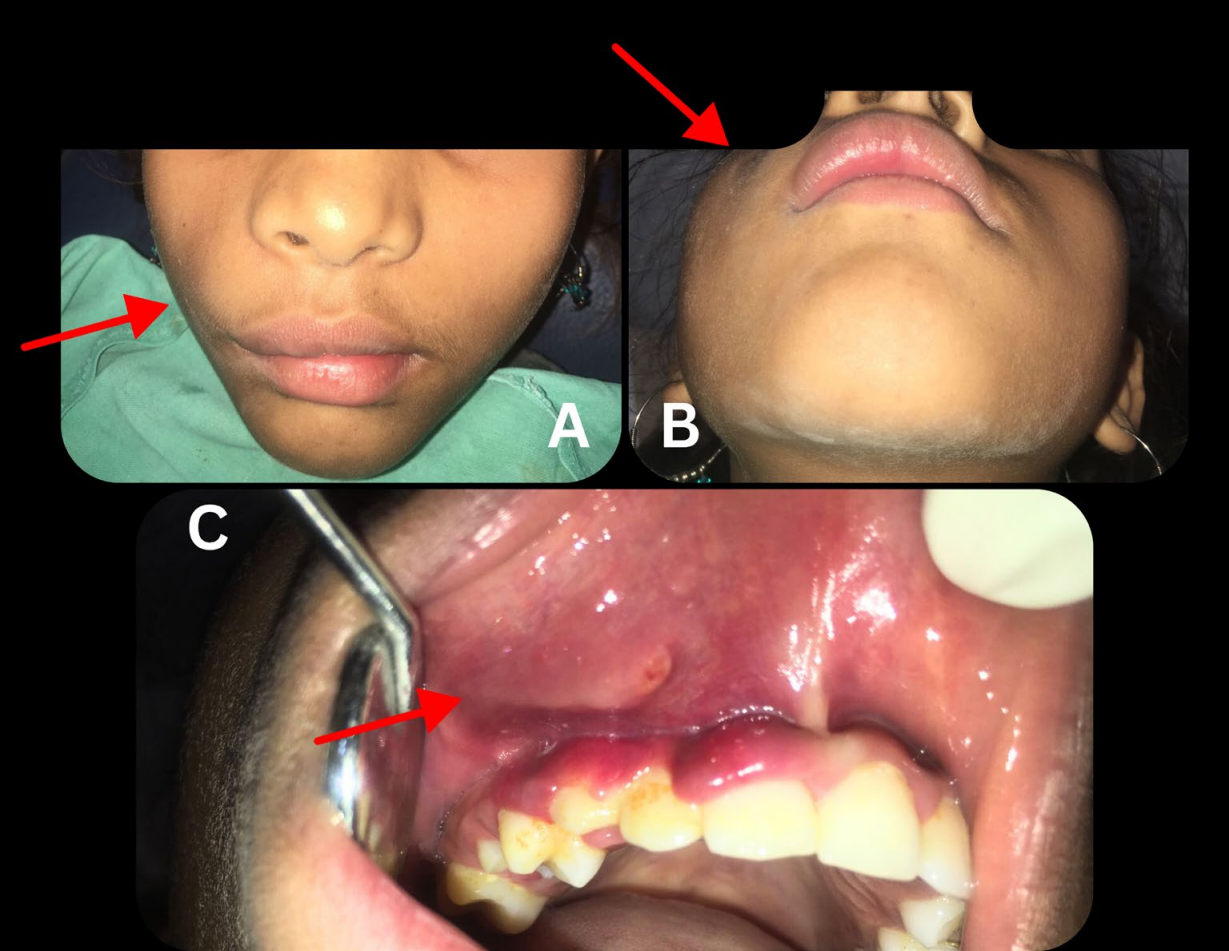
Extraoral and intraoral photographs taken two weeks after initial treatment, (A) Frontal view and (B) Submento‐vertical view showing considerably reduced facial swelling (C) Intraoral view showing reduced lesion size with diminished bluish hue. The area of interest is indicated by a red arrowhead.

A follow‐up visit was made by the patient two weeks after the initial injection. By the time of this visit, the patient was comfortable with no history of pain. She has taken three tablets of paracetamol. The lesion's bluish hue had considerably diminished (Figure 5C), and the swelling had shrunk comparatively (Figure 5A,B).

There were no new complaints, and the lesion looked less noticeable, suggesting that the sclerotherapy was having some effect. In order to further minimize the extent of the lesion, it was decided to repeat the STS injection, and the same analgesic was prescribed.

### 3.3 Third Visit (1 Month Post Second Treatment)

**FIGURE 6:**
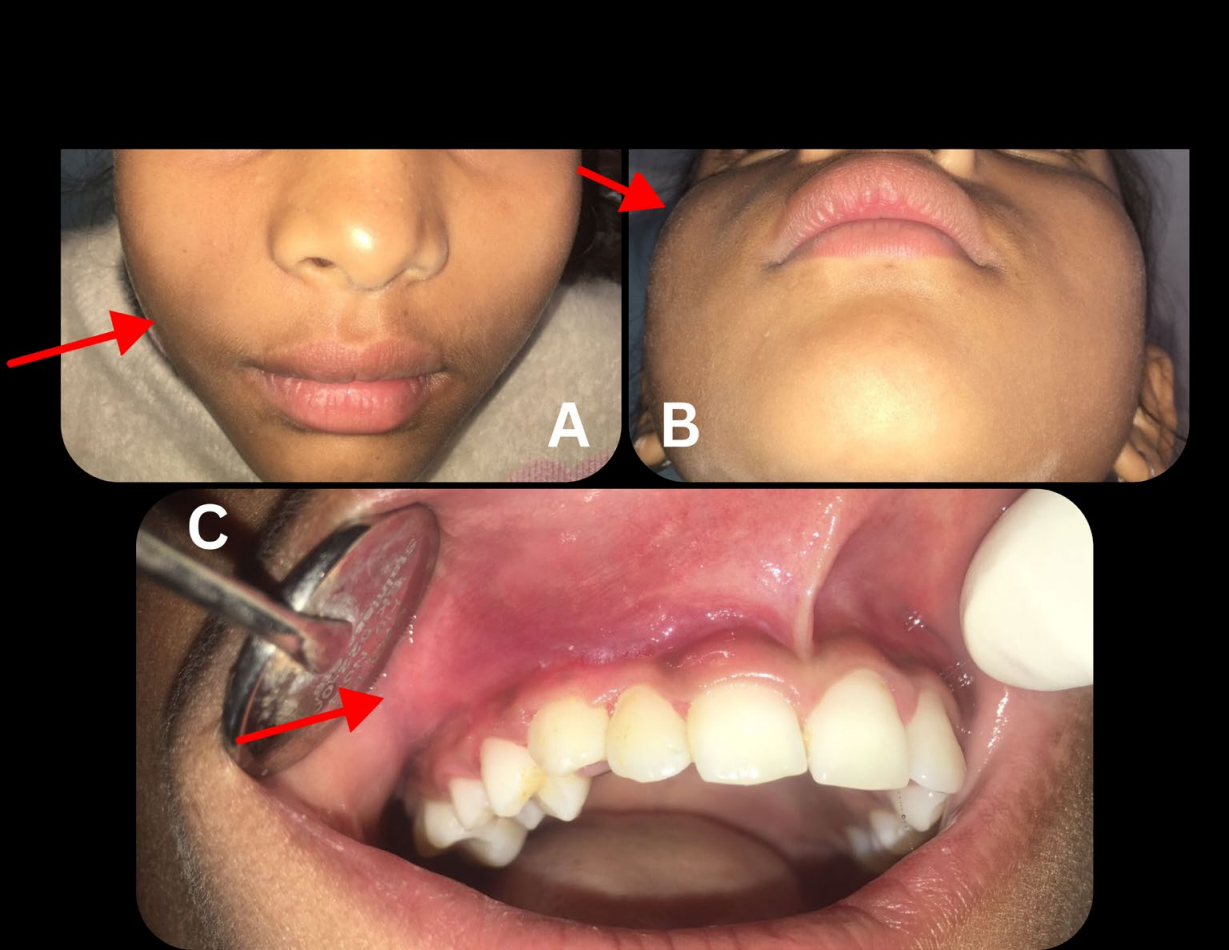
Extraoral and intraoral photographs at the third visit: (A) Frontal view and (B) Submento‐vertical view showing significant reduction in lesion size, and (C) Intraoral view showing absence of bluish hue. The area of interest is indicated by a red arrowhead.

A notable improvement was observed at the third visit, which took place one month after the second injection. There was no longer any bluish tint to the lesion (Figure 6C), which had grown solid and fibrotic. The lesion felt significantly smaller (Figure 6A,B) and was less compressible on palpation, suggesting that the vascular spaces had successfully undergone thrombosis. A third round of injections was given using the same method because the lesion had significantly decreased in size.

### 3.4 Fourth Visit (2‐Week Post Third Treatment)

Additional regression was noted a month later. The lesion seemed more fibrotic and had shrunk in size. Post‐injection, the lesion became firm and reduced to a pea size, indicating a positive response to STS therapy. Another STS session was performed to ensure complete resolution. At this point, no issues were observed, and the patient's parents were instructed to keep gently compressing the area in hopes of seeing even better results.

### 3.5 Fifth Visit (2‐Week Post Fourth Treatment)

The existence of fibrotic tissue was indicated by the lesion's increased reduction in size, firmness, and modest irregularity of texture at the fifth follow‐up visit. The lesion now has the consistency of a little, hard peanut. To guarantee optimum sclerosis of the remaining arterial components, another STS session was conducted. Once more, the patient was instructed to compress the lesion digitally (Finger compression) in order to facilitate the healing process. The parents were instructed to keep an eye on the area for any indications of recurrence or fresh swelling after this visit.

### 3.6 6th, 7th, 8th, and Nine Visit (All Visits Were in One Month Apart)

**FIGURE 7:**
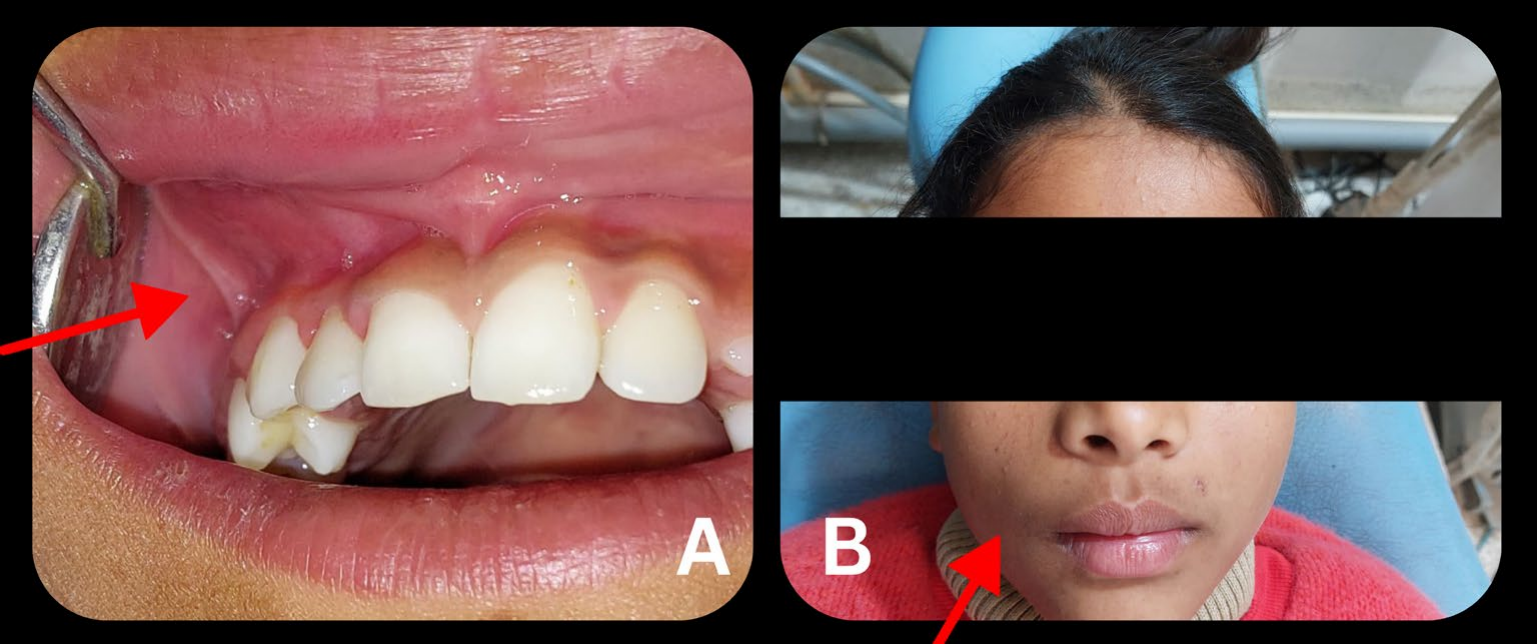
Photographs taken at six‐month follow‐up showing no new symptoms or signs of recurrence: (A) Intraoral view and (B) Frontal view. The area of interest is indicated by a red arrowhead.

The patient did not experience any new symptoms or indications of recurrence in the months that followed (Figure 7A,B). No new alterations were observed at the six‐month follow‐up, and the lesion was stable with little palpable fibrotic tissue. Only a tiny fibrotic mass was left in the lesion, which had greatly shrunk in size nine months following the first therapy. The patient and her parents did not mention any new symptoms.

## 4 Discussion

Oral hemangiomas are challenging because of their propensity for quick growth and can cause both functional and aesthetic problems. Even though the majority of hemangiomas spontaneously involute, lesions that cause symptoms or alter the child's appearance may need to be treated. Surgical excision is still a conventional choice, although it carries a higher risk of scarring and functional impairment, especially in the oral cavity [4, 6].

Sclerotherapy has several advantages over other hemangioma treatments. It is simple, safe, affordable, and widely available because it does not require special equipment or hospitalization. Most importantly, it is highly effective, often leading to partial or complete regression of the lesion without bleeding.

Sclerotherapy has proven effective in treating benign vascular lesions, particularly small ones in cosmetically sensitive areas where surgery might cause undesirable scarring [7].

Frequently used sclerosing agents are sodium morrhuate, sodium psylliate, hypertonic glucose solution, sodium tetradecyl sulfate, ethanolamine oleate, and polidocanol [3, 4, 8].

The amount of sclerosing agent used and the number of treatment sessions depend on the lesion's size, location, and involvement of nearby structures. Results should be assessed before each new dose, typically after a 1‐ to 2‐week interval [4, 7].

The treatment employed in the presented case was sclerotherapy with STS. The concentration of the sclerosing agent (60 mg/2 mL), number of treatment sessions (6 sessions), and intervals between each session (2–4 weeks) were planned. The volume and concentration were supported by established clinical protocols and previous literature for effectively sclerosing oral vascular lesions while minimizing risks, as demonstrated in the successful cases reported by Kim et al. [9] and Alakailly et al. [10], where lesion size remarkably regressed without complications. This dosage was selected based on careful patient evaluation, acknowledging that reactions to the sclerosant agent are different for each patient and that no universal guideline exists for a standard therapeutic dose [9, 10, 11].

The case has proven that intralesional injection of STS was very effective, inducing rapid regression of the lesion after the procedure as only 5 sessions were sufficient for treatment of superficial hemangiomas.

The patient underwent sclerotherapy with STS as the primary treatment modality. The treatment protocol included a sclerosing agent concentration of 30 mg/mL (60 mg/2 mL), five sessions, and intervals of 2 to 4 weeks between sessions, guided by clinical experience and literature on STS for hemangioma management. Intralesional STS injections demonstrated high efficacy, with rapid lesion regression observed after treatment. Successful resolution was achieved in five sessions, and subsequent follow‐up visits, up to nine sessions, confirmed the outcome, with no further injections required after the fifth session, highlighting its suitability for superficial hemangiomas. (Table 1).

**TABLE 1:** Timeline of lesion progression, STS dose, and clinical observations.

Visit	Timepoint	STS Dose Administered	Number of Injection Sites	Observed Clinical Changes	Analgesic Use
Visit 1	Day 0	2 mL (60 mg/2 mL)	4 sites (0.5 mL/site)	Bluish, soft, compressible lesion; no complications post‐procedure	Paracetamol 250 mg TID for 24 h
Visit 2	2 weeks			Bluish hue reduced; swelling decreased in size; no pain	Paracetamol 250 mg TID for 24 h
Visit 3	1 month post‐Visit 2			Lesion firm, no bluish tint, fibrotic areas forming, significant decrease in size of the lesion	Paracetamol as needed
Visit 4	2 weeks post‐Visit 3			Further regression; lesion smaller, firm, pea‐sized	None
Visit 5	2 weeks post‐Visit 4			Harder texture, minor residual swelling; continued fibrotic response	
Visit 6	1 month later	None		Lesion stable; no new growth; no symptoms	
Visit 7	1 month later			Continued regression; minimal palpable lesion	
Visit 8	1 month later			No visible swelling; residual fibrotic nodule	
Visit 9	1 month later (9 months from start)			Lesion completely fibrotic; no signs of recurrence	

With benefits like minimum invasiveness and a low risk of complications, STS sclerotherapy has shown itself to be an effective alternative for treating oral hemangiomas. It causes endothelial injury and clot formation, which causes the lesion to gradually fibrose. Its effectiveness has been demonstrated by numerous studies, which have shown notable decreases in hemangioma size and enhancements in both functional and cosmetic results.

The results of the study were similar to the data of literature relating to sclerotherapy, with variations according to the type of sclerosing agents [3, 4].

Reported potential complications of STS in the literature include pain, edema, ulceration, and rarely ecchymosis and anaphylaxis [3, 4, 10, 11] In the present case, the patient was monitored. Only transient pain was noted initially, which was managed by analgesics. No other local and systemic complications occurred. In line with other published studies [3, 10, 11] this report demonstrates the effectiveness of STS in managing pediatric oral lesions. Although direct comparative evidence with other sclerosing agents such as ethanolamine oleate or polidocanol remains limited [4, 7, 9, 11] the favorable outcome observed here is consistent with prior STS series, which have documented complete or substantial resolution in a majority of cases and also supports the potential of STS as a dependable primary sclerosing agent in such cases.

Despite these advantages, the limitation of STS sclerotherapy must be acknowledged. Efficacy may be reduced in larger and deeper lesions requiring higher doses and often requiring multiple sessions. Additionally, this approach carries the risk of incomplete resolution and lacks histopathological confirmation afforded by surgical excision [3, 10, 11].

Oral hemangiomas have also been investigated for treatment with laser therapy, specifically with pulsed dye lasers (PDL) or Nd:YAG lasers. Compared to standard excision, these methods provide more focused treatment with fewer adverse effects. However, in certain therapeutic settings, laser therapy is less accessible due to its higher cost and need for specialist equipment [12].

Although corticosteroid injections are useful in lowering vascularity and inflammation, they are not recommended for long‐term use due to possible dangers like systemic adverse effects [13].

In general, sclerotherapy combined with STS is a great way to treat children's oral hemangiomas. It is a great option because of its minimally invasive nature and good safety record, especially for lesions that would otherwise necessitate significant surgery.

## 5 Conclusion

Despite being benign, oral hemangiomas can cause serious functional and aesthetic concerns, especially in young individuals. This case report describes how intralesional STS injections were used to successfully treat an oral hemangioma in a 10‐year‐old girl. The procedure prevented the need for invasive surgery by significantly reducing the size of the lesion with no side effects. With encouraging results in terms of lesion regression and aesthetic enhancement, STS sclerotherapy was shown to be a safe, well‐tolerated, and successful treatment.

## Author Contributions

**Niroj Khanal:** conceptualization, project administration, visualization, writing – review and editing. **Bibek Kattel:** conceptualization, visualization, writing – original draft. **Rojeena Adhikari:** conceptualization, writing – review and editing. **Sushma Jaishi:** conceptualization, visualization, writing – original draft. **Pramodman Singh Yadav:** conceptualization, writing – review and editing.

## Consent

Written informed consent was obtained from the patient to publish this report in accordance with the journal's patient consent policy.

## Conflicts of Interest

The authors declare no conflicts of interest.

## Data Availability

The data that support the findings of this study are available from the corresponding author upon reasonable request.
